# SECRET domain of variola virus CrmB protein can be a member of poxviral type II chemokine-binding proteins family

**DOI:** 10.1186/1756-0500-3-271

**Published:** 2010-10-27

**Authors:** Denis V Antonets, Tatyana S Nepomnyashchikh, Sergei N Shchelkunov

**Affiliations:** 1 State Research Center of Virology and Biotechnology "Vector" Rospotrebnadzor, Novosibirsk region, Koltsovo, Russian Federation

## Abstract

**Background:**

Variola virus (VARV) the causative agent of smallpox, eradicated in 1980, have wide spectrum of immunomodulatory proteins to evade host immunity. Recently additional biological activity was discovered for VARV CrmB protein, known to bind and inhibit tumour necrosis factor (TNF) through its N-terminal domain homologous to cellular TNF receptors. Besides binding TNF, this protein was also shown to bind with high affinity several chemokines which recruit B- and T-lymphocytes and dendritic cells to sites of viral entry and replication. Ability to bind chemokines was shown to be associated with unique C-terminal domain of CrmB protein. This domain named SECRET (Smallpox virus-Encoded Chemokine Receptor) is unrelated to the host proteins and lacks significant homology with other known viral chemokine-binding proteins or any other known protein.

**Findings:**

*De novo *modelling of VARV-CrmB SECRET domain spatial structure revealed its apparent structural homology with cowpox virus CC-chemokine binding protein (vCCI) and vaccinia virus A41 protein, despite low sequence identity between these three proteins. Potential ligand-binding surface of modelled VARV-CrmB SECRET domain was also predicted to bear prominent electronegative charge which is characteristic to known orthopoxviral chemokine-binding proteins.

**Conclusions:**

Our results suggest that SECRET should be included into the family of poxviral type II chemokine-binding proteins and that it might have been evolved from the vCCI-like predecessor protein.

## Introduction

Chemokines form a large family of chemoattractant cytokines with low molecular weight (~7-14 kDa) and their sequence similarity range from 20 to 90%. More than 50 distinct chemokines and over 19 different chemokine receptors were discovered to date. All known cellular chemokine receptors are type III transmembrane proteins associated with G-proteins. According to the arrangement of conservative N-terminal cysteine residues chemokines are divided into CC-, CXC-, C- and CX3C-chemokines [1]. Chemokines play important roles in regulation of innate as well as acquired immunity orchestrating leukocyte migration. Through binding to cell surface or intercellular matrix glycosaminoglycans (GAGs) chemokines form chemotactic gradient attracting leukocytes to the sites of injury and inflammation. Chemokines are also involved in embryonic development, organogenesis and other processes [1,2]. Despite low sequence identity between different chemokines all of them share remarkably similar tertiary structure with elongated N-terminal loop followed by three β-chains folded in a "greek key" moiety [3].

During long-lasting coevolution with their hosts poxviruses have developed efficient mechanisms to evade host immune reactions initiated in response to infection. In particular, one of the most important viral immunomodulatory strategies are viroceptors - virally encoded proteins secreted from infected cells - which bind and inhibit biological activity of tumour necrosis factor (TNF), different kinds of interferons, interleukine 18, chemokines and other mediators of host immune system [4-6]. Such viroceptors may be non-homologous to cellular proteins with correspondent biological activity, as it was shown for poxviral chemokine-binding proteins [7]. Some poxviral immunomodulatory proteins possess pleiotropic activity, as it was shown for M-T7 protein of myxoma virus, which is able to bind γIFN as well as chemokines [8]. Recently additional biological activity was discovered for orthopoxviral CrmB and CrmD proteins. Previously known only as TNF-binding proteins, they were shown to bind with high affinity and inhibit CCL25, CCL28, CCL25, CXCL12β, CXCL13 and CXCL14 chemokines. Chemokine-binding activity of CrmB and CrmD proteins was shown to be mediated by their unique C-terminal domain lacking substantial homology to any other known proteins. This domain was named SECRET - Smallpox virus-Encoded Chemokine Receptor [9].

Since C-terminal domain of VARV-CrmB lacks substantial sequence homology with other proteins (excepting orthologous proteins of other orthopoxviruses), and since its spatial structure is not known, we decided to predict it to find related proteins sharing similar structural features with VARV-CrmB SECRET domain. Predicted spatial structure of VARV CrmB SECRET domain was found to be closely related to cowpox virus vCCI protein, belonging to the family of poxviral type II chemokine-binding proteins.

## Results and Discussion

**Figure 1:**
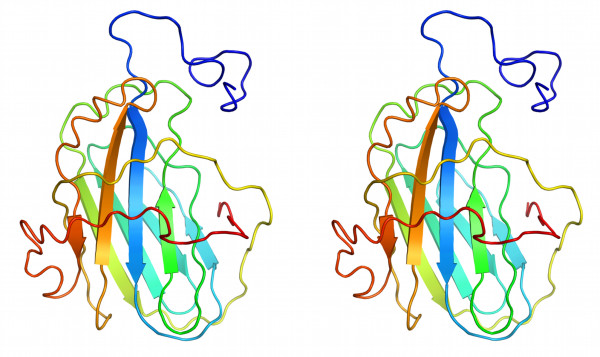
**Stereoscopic view of predicted spatial structure of the SECRET**. Spatial structure of the SECRET domain predicted by I-TASSER [11] is represented with ribbon diagram. Blue color indicates the N-terminus of the SECRET, and its C-terminus is shown in red. Image was produced using PyMOL [21].

Using PSIPRED server [10] VARV-CrmB SECRET domain was predicted to consist of twelve β-strands linked by unordered loops, remaining the overall secondary structure of poxviral type II chemokine-binding proteins (vCkBPII). Using I-TASSER web-server [11], which was shown to be the best server for predicting spatial structures of proteins according to results of CASP7 (CASP - Critical Assessment of protein Structure Prediction) and CASP8 competitions [11,12], we obtained the model of VARV-CrmB SECRET domain. SECRET was predicted to be β-sandwich, composed by two parallel β-sheets connected by several loops (Figure 1). The exterior surface of the first β-sheet was hidden from the solvent by two long engirdling loops. The I-TASSER output can be found in Additional file 1.

**Figure 2:**
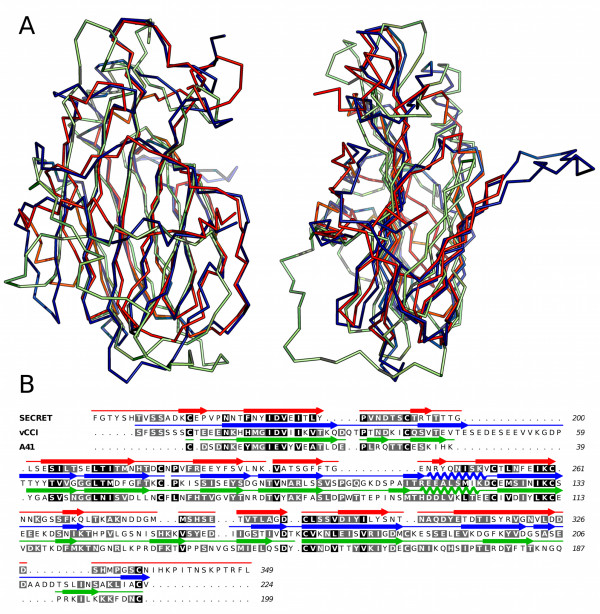
**Superposition of vCCI and A41 structures with modelled SECRET and alignment of their sequences**. (A) Superposition of vCCI, A41 and SECRET was made with CEalign [15]. SECRET is red coloured, vCCI is shown in blue and A41 is green. Left panel shows surfaces formed by β-sheets I of these proteins. Right panel shows the same superposition rotated around y-axis by 90°. Characteristic long extended loop of vCCI is absent in A41 as well as in the SECRET. (B) Alignment of vCCI, A41 and SECRET amino acid sequences produced with structural alignment server TM-align [16]. Alignment was manually edited taking in mind their secondary structures (shown above corresponding sequences) using Aline software [22]. Numbering of amino acid residues in SECRET corresponds to that of full length VARV-CrmB protein (P34015). Gaps are shown as dots, sequences are coloured by amino acid similarity. Amino acid residues of vCCI and A41 are numbered according to numeration found in 1CQ3 and 2VGA PDB files, respectively.

As the best template for modelling SECRET spatial structure I-TASSER server has chosen the structure of CPXV vCCI protein [PDB: 1CQ3] belonging to the family of poxviral type II chemokine-binding proteins which are encoded by almost all known members of *Orthopoxvirus *and *Leporipoxvirus *genera. These abundantly secreted during the early stages of infection 35 kDa glycoproteins lack any homology to cellular chemokine receptors as well as to other known proteins [3,13]. This protein family also includes VACV A41 protein and its orthologs [14]. Despite low sequence identity (~20%) VACV A41 [PDB:2VGA] and CPXV vCCI [PDB:1CQ3] proteins share remarkably similar tertiary structure [14]. We calculated superposition of vCCI and A41 spatial structures with predicted tertiary structure of the SECRET domain (Figure 2A). Superpositions were made using CEalign [15]. Root mean square deviation (RMSD) between coordinates of aligned Cα atoms of SECRET and vCCI was about 2.33 Å (identity between structurally aligned amino acid residues was 14.2%), RMSD between coordinates of aligned Cα atoms of SECRET and A41 was 3.28 Å (10.8% of identity), RMSD between coordinates of aligned Cα atoms of A41 and vCCI was about 3.06 Å (19.9% of identity). The sequences of these proteins were aligned using TM-align [16] server and then manually edited according to their either predicted (SECRET) or known (vCCI, A41) secondary structures (Figure 2B). According to produced alignment SECRET shared 16.3% of sequence identity with vCCI and 15.3% with A41 protein; sequence identity of vCCI to A41 was about 25.5%.

Although spatial structures of vCkBPII proteins are remarkably similar, different members of the family have notable characteristic structural differences. Thus VACV A41 lacks long extended loop which connects the 2^nd ^and the 3^d ^β strands of vCCI (Figure 2A). This loop is conserved in vCCI and its orthologs and was shown to make these proteins able to bind wide spectrum of CC-chemokines with high affinity [3,13,17]. Comparison of known tertiary structure of VACV A41 with predicted spatial structure of the SECRET domain of VARV CrmB revealed that SECRET probably also lacks this loop (Figure 2). This finding gives a bit of support to hypothesis of Ruiz-Arguello and his colleagues [14] that high selectivity and similar specificity towards chemokines, observed for VACV A41 and ectromelia virus (ECTV) E163 protein (orthologous to A41) and SECRET-domain containing proteins, should be underlaid by common structural traits of these vCkBPs. VACV A41 and ECTV E163 bind with high affinity CCL21, 25, 26, 28, CXCL12α, CXCL12β, CXCL13 and CXCL14 chemokines [3,14]; and SECRET domains of VARV CrmB, ECTV CrmD and some other SECRET-containing proteins were shown to bind with high affinity CCL25, 28, CXCL12β, CXCL13 and CXCL14 chemokines [9].

**Figure 3:**
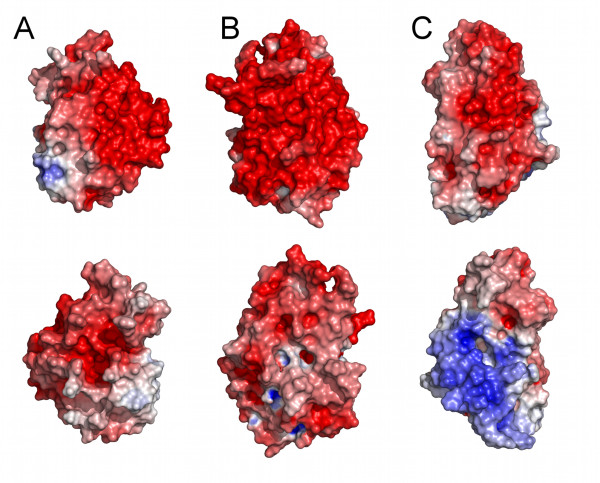
**Electrostatic potential surfaces of SECRET (A), vCCI (B) and A41 (C)**. Upper panel shows chemokine-binding surfaces formed by β-sheets II of corresponding molecules. Lower panel shows the same molecules rotated around y-axis by 180°. Electrostatic potential was calculated with DelPhi software [20]. Negatively charged area is coloured in red and blue colour indicates positive electrostatic charge. Image was produced using PyMOL [21].

Furthermore, although in all known vCkBPs exterior surface of the second β sheet has pronounced electronegative potential due to multiple acidic amino acid residues (Figure 3A) that is favourable to the interaction with positively charged conservative patches of basic amino acid residues found in all known chemokines [3,7,13,17], charge distributions of the opposite surfaces of different vCkBPs may have remarkable distinctions [3,18] (Figure 3B). For example, in vCCI and in the majority of its orthologs this surface has weak electrostatic charge, whereas corresponding surfaces of A41 and its orthologs have pronounced positive charge making these proteins able to bind negatively charged glycosaminoglycans (GAGs) [3]. Myxoma virus M-T1 protein was also shown to bind GAGs, and unlike the majority of vCCI orthologs it has prominent positive electrostatic potential on the surface formed by β-sheet I encircled by two loops [18]. Thus ability to bind GAGs makes certain vCkBPs capable of blocking leukocyte influx into the site of viral replication interfering with chemotactic gradient formation through inhibiting interaction of chemokines with cell surface and extracellular matrix GAGs [3,18,19]. Other vCkBPs, which could not bind GAGs, realize their biological activity only through competing with cellular chemokine receptors for chemokines binding. As predicted by us VARV CrmB SECRET domain also has prominent electronegative potential on the surface formed by β-sheet II, but its opposite surface have no positively charged areas (Figure 3). Thus VARV CrmB SECRET domain is likely unable to bind GAGs and should realize its biological activity only through its ability to interfere with binding of certain chemokines to their cellular receptors that was discovered by Alejo and his colleagues [9].

## Conclusions

Our prediction that VARV CrmB SECRET domain belongs to the family of poxviral type II chemokine-binding proteins is supported by the following: 1. SECRET was shown to bind several chemokines with high affinity [9]; 2. its ligand-binding surface was predicted to have prominent electronegative charge required for binding to positively charged conservative amino-acid residues of chemokines, as it was shown for other members of vCkBPsII [3,7,13,17]; 3. its selectivity towards chemokines is likely to be associated with lacking long extended negatively charged loop (present in vCCI and its orthologs [3,7,13,17]) as it was shown for A41 protein [3]. Thus the predicted structural similarity of SECRET with other vCkBPsII indicates that despite low similarity of their sequences they were most likely derived from the common ancestor. It's curiously that in genomes of *Leporipoxvirus *and *Orthopoxvirus *genera members genes coding for CrmB orthologs are situated in immediate proximity to the genes coding for vCCI orthologs, and we think, it would be of interest to examine phylogenetic relations between the SECRET domain of CrmB and vCCI orthologs.

## Methods

Prediction of secondary structure of VARV-CrmB SECRET domain was done using PSIPRED server [10]. Spatial structure of SECRET domain was modeled using I-TASSER web-server [11]. The server selected the structure of cowpox virus (CPXV) vCCI (viral CC-chemokine inhibitor) protein [PDB:1CQ3] as the best template for modelling SECRET domain. Electrostatic potential of molecular surfaces of all compared proteins was calculated using DelPhi software [20]. Superpositions of the structures were made using CEalign [15]. All molecular graphic images were produced using PyMOL [21]. VARV-CrmB amino acid sequence used in this work was taken from [Swiss-Prot:P34015]. Amino acid sequences of CPXV vCCI [PDB:1CQ3], vaccinia virus (VACV) A41 chemokine-binding protein [PDB:2VGA] and VARV-CrmB SECRET domain were structurally aligned using TM-align server [16]. Alignment was manually edited taking in mind the secondary structures (shown above corresponding sequences) of the proteins using Aline software [22].

## Competing interests

The authors declare that they have no competing interests.

## Authors' contributions

DVA designed the concept of the study, submitted the modelling task to I-TASSER, analyzed produced models and drafted the manuscript. TSN has produced molecular images and alignments. She was also involved in drafting and writing the manuscript. SNS has been involved in writing the manuscript and in its critical revision and has given the final approval for the version to be published. All authors read and approved the final manuscript.

## Supplementary Material

Additional file 1 **Output from I-TASSER web-server**. This file contains the output from I-TASSER web-server including all generated models and alignments. Click here for file
