# Supplementary material for: SECRET domain of variola virus CrmB protein can be a member of poxviral type II chemokine-binding proteins family
Source: BMC Res Notes. 2010 Oct 27;3:271. doi: 10.1186/1756-0500-3-271 (PMC2987869; doi:10.1186/1756-0500-3-271)
Supplement: Additional file 1 — Output from I-TASSER web-server. This file contains the output from I-TASSER web-server including all generated models and alignments. [file 1756-0500-3-271-S1.ZIP › I-TASSER results.htm]

I-TASSER results

[Home]
[Server]
[Queue]
[About]
[Remove]
[Statistics]
  

|  |
| --- |
| I-TASSER results for job id S28327 |
| |  | | --- | | Submitted Sequence |   |  |  | | --- | --- | | >SECRETdVARV FGTYSHTVSSADKCEPVPNNTFNYIDVEITLYPVNDTSCTRTTTTGLSESILTSELTITM NHTDCNPVFREEYFSVLNKVATSGFFTGENRYQNISKVCTLNFEIKCNNKGSSFKQLTKA KNDDGMMSHSETVTLAGDCLSSVDIYILYSNTNAQDYETDTISYRVGNVLDDDSHMPGSC NIHKPITNSKPTRFL | | |
| |  | | --- | | Predicted Secondary Structure |   |  |  | | --- | --- | | **Sequence** | 20                  40                  60                  80                 100                 120                 140                 160                 180                    |                   |                   |                   |                   |                   |                   |                   |                   |                 FGTYSHTVSSADKCEPVPNNTFNYIDVEITLYPVNDTSCTRTTTTGLSESILTSELTITMNHTDCNPVFREEYFSVLNKVATSGFFTGENRYQNISKVCTLNFEIKCNNKGSSFKQLTKAKNDDGMMSHSETVTLAGDCLSSVDIYILYSNTNAQDYETDTISYRVGNVLDDDSHMPGSCNIHKPITNSKPTRFL | | **Prediction** | CCCCCCCCCCCCCSSSCCCCCSSSSSSSSSSSSCCCCSSSSSCCCCCCSSSSSSSSSSSSSCCCCCCCCCCCSSSSCCCSSSSSSSCCCCSSCCCCSSSSSSSSSSSCCCCCCCSSCCCCCCCCCCCCCCCSSSSSSSCCCCCSSSSSSSCCCCCCCSCSSSSSSSCSSSSCCCCCCCCCCCCCCCCCCCCCCCC | | **Conf.Score** | 962255337653015457885278998889875576424265358852589986799998547898021101467604478741361774335731379999999967999730354323378733555524899852244746999994489877040169999701681388799853302222368975449 | |
| |  | | --- | | Top 5 Models predicted by I-TASSER |   |  |  |  |  |  | | --- | --- | --- | --- | --- | |  |  |  |  |  | | Download Model 1 | Download Model 2 | Download Model 3 | Download Model 4 | Download Model 5 | | C-score=-2.08 | C-score=-2.77 | C-score=-2.62 | C-score=-2.75 | C-score=-4.74 | | (more about C-score of generated models) | | | | | |
| |  | | --- | | Top 10 templates used by I-TASSER |   |  |  |  |  |  |  |  |  |  | | --- | --- | --- | --- | --- | --- | --- | --- | --- | | Rank | PDB Hit | Iden1 | Iden2 | Cov. | Norm. Z-score | Download Align. |  | 20                  40                  60                  80                 100                 120                 140                 160                 180                    |                   |                   |                   |                   |                   |                   |                   |                   | | |  | | | | | | | Sec.Str Seq | CCCCCCCCCCCCCSSSCCCCCSSSSSSSSSSSSCCCCSSSSSCCCCCCSSSSSSSSSSSSSCCCCCCCCCCCSSSSCCCSSSSSSSCCCCSSCCCCSSSSSSSSSSSCCCCCCCSSCCCCCCCCCCCCCCCSSSSSSSCCCCCSSSSSSSCCCCCCCSCSSSSSSSCSSSSCCCCCCCCCCCCCCCCCCCCCCCC FGTYSHTVSSADKCEPVPNNTFNYIDVEITLYPVNDTSCTRTTTTGLSESILTSELTITMNHTDCNPVFREEYFSVLNKVATSGFFTGENRYQNISKVCTLNFEIKCNNKGSSFKQLTKAKNDDGMMSHSETVTLAGDCLSSVDIYILYSNTNAQDYETDTISYRVGNVLDDDSHMPGSCNIHKPITNSKPTRFL | | 1 | 1cq3A | 0.13 | 0.26 | 0.97 | 1.11 | Download |  | SFSSSSSCTEEENKHQTPTNDKICQSVTEVTESEDESEEVVKGDPTTYYTVVGGGLTMDFGFTKCPKISSISEYSDGNTVNARLSSVSPGQGKDSPAICEMSINIKCSEEEKDSNIKTHPVLGSNISHKKVSTIVDTKCVKNLEISVRIGDMCKESEVKDGFKYVDGSASEDAADDTSLINSAKLIA------CV | | 2 | 1cq3A | 0.18 | 0.26 | 0.95 | 1.27 | Download |  | ------SFSSSSSCTEEENKHHMGIDVIIKVTPTNDKICQSVTEVTTYYTVVGGGLTMDFGFTKCPKISSISEYSDGNTVNARLSAITREEALSMIKDCEMSINIKCSEEEKDSNIKTHPVLGSNISHKKGSTIVDTKCVKNLEISVRIGDMCKE---SSELEVKDGFKYVDGSASEDAADDTSLINSAKLIACV | | 3 | 1nt0A | 0.06 | 0.17 | 0.96 | 1.09 | Download |  | EPVFPEKYGNHQDRSTLTAPPGFRLRLYFTHFNLELS---YRCEYDFVKLTSGTKVLATLCGQESTDTERNDTFYSLGPSLKVTFHSDY----PFTGFEAFYAAEDVDECRPCDHYCHYLGGYYQPYPKLSSCAYNIRLEEGFSITLDFVESFDVEMHCDSLKIEYGPFCGKTLPPRIETDSNKVTTDESGNHTA | | 4 | 1b12A | 0.09 | 0.20 | 0.96 | 1.02 | Download |  | FIYEPFQIPSGSMMPTLLIGDFILVEIETGHPKRGDIVVFKYPEDPKLDYIKRAVGKVTYDPVSK-------ELTIQPGCSSGQACENALPVTYSNVEPSDFVQTFSRRNGGEATSGFFEVPKNETKENGIRLSERKETLGDVTHRILTVPYQQPGQQLATWIVPPGQYFMMGDNRDNSADSRDGLRLSRIGGIH | | 5 | 1cq3A | 0.18 | 0.26 | 0.93 | 0.73 | Download |  | ------SFSSSSSCTEEENKHHMGIDVIIKVTKQNDKICQSVTEVDPTTYYTVVGLTMDFGFTKC-PKISSISEYSDGNTVNARLSQGKDTREEMIKDCEMSINIKCSEEEKDSNIKTHPVLGSNISHKKVGSTIVDTCVKNLEISVRIGDKESSEEVKDGFKYVDGSASE------DAADDTSLINSAKLIACV | | 6 | 1cq3A | 0.20 | 0.26 | 0.91 | 0.64 | Download |  | ------SFSSSSSCTEEENKHHMGIDVIIKVTPTNDKICQSVTEVDPYYTVVGGGLTMDFGFTKC-PKISSISEYSDGNTVNARLSSVPGQGKDMIKDCEMSINIKC-SEEEKDSNIKTHPVLGSNISHKKVTIVDTKCVKNLEISVRIGDMCKELEVKDGFKYVDGSASEDDTSLINSA---KLIACV------ | | 7 | 1cq3A | 0.20 | 0.26 | 0.93 | 0.65 | Download |  | ------SFSSSSSCTEEENKHHMGIDVIIKVTPTNDKICQSVTEVTESYTVVGGGLTMDFGFTKC-PKISSISEYSDGNTVNARLGQGKDS-PAITKDCEMSINIKCSEEEKDSNIKTHPVLGSNISHKKVSTIVDTKCVKNLEISVRIGDMCKESEVKDGFKYVDGSASEDAADDTSLINSAKLIACV------ | | 8 | 1k32A | 0.12 | 0.20 | 1.00 | 0.96 | Download |  | MPNLLHDLKSGSTRKIVSNLGVINNTADLYFYNGENGEIKRITYFSGKSTGRRMFTDVAGFDPDGNNLGPATHILFADGRRVIGRNTFELPHWKGYRGGTRGKIWIEVNSGAFKKIVDMSTHVSSPVIVGHRIYFITDIDGFGQIYSTDLDGKDLFTDYYPRHLNTGSIYIFNPDTEKIEKIEIGDLESPEDRII | | 9 | 1cq3A | 0.19 | 0.26 | 0.94 | 0.85 | Download |  | ------SFSSSSSCTEEENKHHMGIDVIIKVTKQDQTSVTEVTESEDEYTVVGGGLTMDFGFTKCPKISSISEYSDGNTVNARLSSVSPGQGKDSPKDCEMSINIKCSEEEKDSNIKTHPVLGSNISHKKVSTIVDTKCVKNLEISVRIGDMCKESSELDGFKYVDGSASEDAADDTSLINSAKLIACV------ | | 10 | 1cq3A | 0.19 | 0.26 | 0.90 | 0.72 | Download |  | ------SFSSSSSCTEEENKHHMGIDVIIKVTPTNDKICQSVTKGDPYYTVVGGGLTMDFGFTKC-PKISSISEYSDGNTVNARLSSVSGQGKDMIKDCEMSINIKC-SEEEKDSNIKTHP---VLGSNISKKVVDTKCVKNLEISVRIGDMCKELEVKDGFKYVDGSASEDDTSLIN---SAKLIACV------ | | |  |  | | --- | --- | | (a) | All the residues are colored in black; however, those residues in template which are identical to the residue in the query sequence are highlighted in color. Coloring scheme is based on the property of amino acids, where polar are brightly coloured while non-polar residues are colored in dark shade. (more about the colors used) | | (b) | Rank of templates represents the top ten threading templates used by I-TASSER. | | (c) | Ident1 is the percentage sequence identity of the templates in the threading aligned region with the query sequence. | | (d) | Ident2 is the percentage sequence identity of the whole template chains with query sequence. | | (e) | Cov. represents the coverage of the threading alignment and is equal to the number of aligned residues divided by the length of query protein. | | (f) | Norm. Z-score is the normalized Z-score of the threading alignments. Alignment with a Normalized Z-score >1 mean a good alignment and vice versa. | | (g) | Download Align. provides the 3D structure of the aligned regions of the threading templates. | | (h) | The top 10 alignments reported above (in order of their ranking) are from the following threading programs: | |  | 1: PROSPECT2   2: FUGUE   3: PROSPECT2   4: PROSPECT2   5: MUSTER   6: HHSEARCH   7: SP3   8: PROSPECT2   9: PPA-I   10: HHSEARCH I | | | | | | | | | | |
| |  | | --- | | 10 proteins in PDB which are structurally closest to the first I-TASSER model (identified by TM-align) |   |  |  |  |  |  |  |  |  |  |  |  |  |  |  |  |  |  |  |  |  |  |  |  |  |  |  |  |  |  |  |  |  |  |  |  |  |  |  |  |  |  |  |  |  |  |  |  |  |  |  |  |  |  |  |  |  |  |  |  |  |  |  |  |  |  |  |  |  |  |  |  |  |  |  |  |  |  |  |  |  |  |  |  |  |  |  |  |  |  |  |  |  |  |  |  |  |  |  |  |  |  |  |  |  |  |  |  |  |  |  |  |  |  |  |  |  |  |  |  |  |  |  |  |  |  |  |  |  |  |  |  |  |  |  |  |  |  |  |  |  |  |  |  |  |  |  |  |  |  | | --- | --- | --- | --- | --- | --- | --- | --- | --- | --- | --- | --- | --- | --- | --- | --- | --- | --- | --- | --- | --- | --- | --- | --- | --- | --- | --- | --- | --- | --- | --- | --- | --- | --- | --- | --- | --- | --- | --- | --- | --- | --- | --- | --- | --- | --- | --- | --- | --- | --- | --- | --- | --- | --- | --- | --- | --- | --- | --- | --- | --- | --- | --- | --- | --- | --- | --- | --- | --- | --- | --- | --- | --- | --- | --- | --- | --- | --- | --- | --- | --- | --- | --- | --- | --- | --- | --- | --- | --- | --- | --- | --- | --- | --- | --- | --- | --- | --- | --- | --- | --- | --- | --- | --- | --- | --- | --- | --- | --- | --- | --- | --- | --- | --- | --- | --- | --- | --- | --- | --- | --- | --- | --- | --- | --- | --- | --- | --- | --- | --- | --- | --- | --- | --- | --- | --- | --- | --- | --- | --- | --- | --- | --- | --- | --- | --- | --- | --- | --- | | | Rank | TM-score | RMSDa | IDENa | Cov. | PDB Hit | | --- | --- | --- | --- | --- | --- | | 1 | 0.8192 | 2.42 | 0.14 | 0.94 | 1cq3A Model1 | |  | | | | | | | 2 | 0.6240 | 3.39 | 0.11 | 0.79 | 2vgaA Model1 | |  | | | | | | | 3 | 0.4687 | 4.58 | 0.04 | 0.70 | 1uc2A Model1 | |  | | | | | | | 4 | 0.4618 | 4.62 | 0.04 | 0.70 | 2epgA Model1 | |  | | | | | | | 5 | 0.4384 | 4.58 | 0.06 | 0.65 | 1q9jA Model1 | |  | | | | | | | 6 | 0.4338 | 4.69 | 0.03 | 0.66 | 1oacA Model1 | |  | | | | | | | 7 | 0.4314 | 4.70 | 0.04 | 0.66 | 2gvgA Model1 | |  | | | | | | | 8 | 0.4239 | 4.81 | 0.04 | 0.65 | 2e1tA Model1 | |  | | | | | | | 9 | 0.4187 | 5.15 | 0.03 | 0.67 | 2ohyA Model1 | |  | | | | | | | 10 | 0.4165 | 5.19 | 0.03 | 0.66 | 1jmmA Model1 | |  | | | | | | | | Structural alignment using TM-align | | --- | | ------SFSSSSSCTEEENKHHMGIDVIIKVTKQDQTPTNDKICQSVTEVTESEDESEEVVKGDPTTYYTVVGGGLTMDFGFTKC-PKISSISEYSDGNTVNARLSSVSPGQGKDSPAITREEALSMIKDCEMSINIKCSEEEKDSNIKTHPVLGSNISHKKVSYEDIIGSTIVDTKCVKNLEISVRIGDMCKESSELEVKDGFKYVDGS-A-SE-DAADD-TSLINSAKLIA-CV  FGTYSHTVSSADKCEPVPNNTFNYIDVEITLYP---V--NDTSCTRTTTTG---------------LSESILTSELTITMNHTDCNPVFREEYFSVL-NKVATSGFFTG-----ENRYQ---NI-SK--VCTLNFEIKCNNKGSSFKQLTKAKNDDGMMSHS--ET-VT-LAGDC---LSSVDIYILYSNTNAQDY-ETDTISYRVG-NVLDDDSHMPGSCNIHKPITNSKPTRFL | |  | | --------------C-DSDNKEYMGIEVYVEATLDEPLRQTTCESKIHKYGASVSNGGLNISVDLLNCFLNFHTVGVYTNRDTVYAKFASLDPWTTEPINSMTHDDLVKLTEECIVDIYLKCEVDKTKDFMKTNGNRLKPRDFK--------------TVPPSNVGSMIELQSDYCVNDVTTYVKIYDECGNIKQHSIPTLRDYFT---TKN----G-QPRKI--LKKKFD-N-C-  FGTYSHTVSSADKCEPVP-NNTFNYIDVEITLYP--VNDTSCTRTTTTGLSESILTSELTITMNHTDCNPVFREEYFSVLNKVATSGFFTG-EN----RYQN---IS--K--VCTLNFEIKCNNK--GS---------------SFKQLTKAKNDDGMMSHSE--T--VTLAGDC-LSSVDIYILYSNTNAQ----DYETDTISYRVGNVLDDDSHMPGSCNIHKPITNSKPTRFL | |  | | VVPLKRIDKIRWEIPKFDKRMRVPGRVYADEVLLEKMKNDRTLEQATNVAMLPGIYKYSIVMPDG---------HQGYGFPIGGVAAFDVKEGVISPGGIGYDINCGVRLIRTNLTEKEVRPRIKQLVDTLFKNVPSGVGSQGRIKLHWTQIDDVLVDGAKWAVDNG----------YGWERDLERLEEG-GRMEGADPEAVSQRAKQRGAPQLGSLGSGNHFLEVQVVDKIFDPEVAKAYGLFEGQVVVMVHTGSRGLGHQVASDYLRIMERAIRKYRIPWPDRELVSVPFQSEEGQRYFSAMKAAANFAWANRQMITHWVRESFQEVFKQD------------------------PEGDLGMDIVYDVAHNIGKVEEHEVDGKRVKVIVHRKGA-TRAFPPGHEA------------VPRLYRDVGQPVLIPGSMGTASYILAGTEGAMKETFGSTCHGAGRVLSRKAATRQYRGDRIRQELLNRGIYVRAASMRVVAEEAPGAYKNVDNVVKVVSEAGIAKLVARMRPIGVAKG  -----------------------------------FG----------------------------TYSHTVSSADKCEPV---------------PNN-TF--N-YIDVEITLYPV--ND--TS---CTR-T--TT-----TGLS----------------------ESILTSELTITMN----------HTDC---------------N---P--VF---REEYFSVL----------------NKVATSGFFTGEN--R-YQ--------------------------------------NISK--V--CT-LN--FE--IK--CNNKG--SSFKQLTKAKNDDGMMSHSETVTLAGDCLSSVDIYILYSN-------------------TNAQDYETD------TISYRVGNVLDDDSHMP--G-SCNIH--------KP------------------------------------------------------------------------------------ITNSKPTRFL | |  | | FFEKIAPYTYRIPRQGKRVDAVFFASKEILKDLEAENYASLQQLNVATLPGIVEPALAPDI-----------HWGYGFPIGGVAAFDPEEGGVVSPGGVGFDINCGVRLLASHLTLEDLLPRQKELADALYRLVPSRDVRFSKRELKEILKEGAGWLVKRG----------YGYPEDVRFIESQ-GRLPWANPDKVSERAFERGAPQIGTLGSGNHFLEVQYVDEVYDEEAALAFGLFKGQVTVLIHTGSRGLGHQVCQDYVERFLKVAPRYGIELVDKQLAAAPIKSPEGQDYLQAAAAANFAFANRQLIAHFVREAFEKVGF-------------------------TPRDHGLRVLYDLAHNNAKFEEHRGRRVLVHRKGA-TRAFGPGHPE------------VPEEYRRVGQPVLVPGDGRYSYVLAGTEKAEVSFGSSCHGAGRNLVKELAERGILVRAAVSLVVEAVEGAGIGKKVARLRPLIVVKG  -------------------------------------------------------------FGTYSHTVSSADKCEPV---------------PNN--TF--N-YIDVEITLYP---VND-TS---CT-RT--TT-TGLS---------------------ESILTSELTITMN----------HTDC---------------N---P-VF----REEYFSVL----------------NKVATSGFFTGEN--RYQN--------------------------------------ISK--V--CT--LN-FE--IK-CNNKG-SSFKQLTKAKNDDGMMSHSETVTLAGDC-LSSVDIYILYSN---------------TNAQDYETD------TISYRVGNVLDDDSHMPG---SCNIH-------KP------------------------------------------------------ITNSKPTRFL | |  | | MFPGSVIRKLSHSEE----------------VF-AQYEVFTSMTIQLRGV-IDVDALSDAFDALLETHPVLASHLEQSSDGGWNLVADDLLHS-GICVIDAELRLDQSV-------------SLLHLQLILREGGAELTLYL-H---HCMA--DGHHGAVLVDELFSRYTDAVTTGDPGPITPQPTPLSMEAVLAQRGIRKAERFMSVMYAYPG-----------LPQAVPVTRLWLSKQQTSDLMAFGREHRLSLNAVVAAAILLTEWQLRNTPHVPIPYVYPVDLRFVLAPPVAPTEATNLLGAASYLAEIGPNTDIVDLASDIVATLRADLANGVIQQSGLHFGTAFEGTPPGLPPLVFCTDATSFPTMRTPPGLEIEDIKGQFYCSISVPLDLYSCAV-----YAGQLI-IEHHGHIAEPGKSLEAIRSLLCTVPSEYG---------------  ---------------FGTYSHTVSSADKCEPV-PNNTFNYIDVEITLYPVNDTS--CTR-TT--T-TGLSE----------------------SILTS-----------ELTITMNHTDCNPVFREEYFSVL-NKVATSGFFTGENRYQNISKVCT--LN-FE--IK--C--NNKG--------------------------------------SSFKQLTKAKNDDGMMSHSE-----------------------------------------------------------------------------------------------------------------------------------TVTLA-GDC---LSSVDIYILYSNTNAQDYETDTISYRVGNVLDDDSHMPGSC-------------------------NIHKPITNSKPTRFL | |  | | AHMVPMDKTLKEFGADVQWDDYAQLFTLIKDGAYVKVKPGAQTAIVNGQPLALQVPVVMKDNKAWVSDTFINDVFQSGLDQTFQVEKRPHPLNALTADEIKQAVEIVKASADFKPNTRFTEISLLPPDKEAVWAFALENKPVDQPRKADVIMLDGKHIIEAVVDLQNNKLLSWQPIKDAHGMVLLDDFASVQNIINNSEEFAAAVKKRGITDAKKVITTPLTVGYFDGKDGLKQDARLLKVISYLDVG------DGNYWAHPIENLVAVVDLEQKKIVKIEEGPVVPVPMTARPFDGRDRVAPAVKPMQIIEPEGKNYTITGDMIHWR---------------------NWDFHLSMNSR---VGPMISTVTYNDNGTKRKVMYEGSLGGMIVPYGDPDIGWYFKAYLDSGDYGMGTLTSPIARGKDAPSNAVLLNETIADYTGVPMEIPRAIAVFERYAGPEYKHQEMGQPNVSTERRELVVRWISTVGNDYIFDWIFHENGTIGIDAGAT-GI-EAVKGVKAKTMHDETAKDDTRYGTLIDHNIVGTTHQHIYNFRLDLDVDGENNSLVAMDPVVKPNTAGGPRTSTMQVNQYNIGNEQDAAQKFDPGTIRLLSNPNKENRMGNPVSYQIIPYAG----------------GTHPVAKGAQFAPDEWIYHRLSFMDKQLWVTRYHPGERFPEGKYPNRSTHDTGLGQYSKDNESLDNTDAVV-WMTTGTTHVARAEEWPIMPT-------------------------EWVHTLLKPWNFFDETPTLGALK--------  --------------------------------------------------------------------------------------------------------------------------------------------------------------------------------------------------------------------------------------------------------FGTYSHTVS--S---------------------------------ADK--------------------------------CEPVPNNTFNYIDVEITLYPVNDTSCTRTTTTGLSESILTS-E-------------LTITMNHT-------------DCNP-------------------------------------------------------------------------------V--FREEYFSVL--NKVATSGFFTGENRYQN-----------------------------ISKVCTLNFEIKCN---------------------------------------------------------------------------NKGSSFKQLTKAKNDDGMMSHSET----------------V-TLAGD-------------------------------------CLSSVDIYILYSNTNAQD------YETDTISYRVGNVLDDDSHMPGSCNIHKPIT-----------------NSKPTRFL | |  | | FNILLATDS-YK-VTHYKQYPPNTSKVYSYFE----------------C--REYEETVFYGLQYILNKYLKGKVVTKEKIQEAKDVYKEHFQDDVFNEKGWNYILEKYDGHLPIEIKAVPEG--FVIPRGNVLFTVENTDPECYWLTNWI---ETILVQSWYPITV------ATNSR----------------------EQKKILAKYLLETSGNLDGLEYKLHDFGYRGVSSQETAGIGASAHLVNFKGTDTVAGLALIKKYYGTKDPVPGYSVPAAEHSTITAWGKDHEKDAFEHIVTQFSSVPVSVVSDSYDIYNACEKIWGEDLRHLIVSRSTQAPLIIRPDSGNPLDTVLKVLEILGKKFPVTKGYKLLPPYLRVIQGDGVDINTLQEIVEGMKQKMWSIENIAFGSGGGLLQKLTRDLLNCS---------------------------FKCSYVVTN-GL-GINVFKDPVADPNKRSKKGRLSLHRTPAGNFVTLEEGKGDLEEYGQDLLHTVFKNGKVTKSYSFDEIRKNAQLNI-------  --FGTYSHTVSSADKCEPVPN--N-TFNYIDVEITLYPVNDTSCTRTTTTGLSESILT----------------------------------------------------------------SELTITMNHT------------------DCNPV--FREE-YF--SVLNKVAT---SGFFTGENRYQNISKVCTLNFEIKCNNK----------------GSSF-K---Q----L-TKA--------------------------------------------------------------------------------------------------------------------------------------------------------------------------------------------KNDDGMMSHSETVTLAGDCLSSVDIYILYSNTNA--QD-YE-----------------TDTISYRVGNVLDD----------DSHMPGSCNI------HKPITN-----------SKPTRFL | |  | | ILTVLEQSQVSPPPDTLGDKSLQLTFFDF------------------FWLRSPPINNLFFYELPITRSQFTETVVPNIKHSLSITLKHFYPFVGKLVVYPAPTKKPEICYVEGDSVAVT-FAECNLDLNELTGNHPRNCDKFYDLVPILGESTRLSDCIK------------IPLF-SVQVTLFPNQGIAIGITN-H-----HCLGDASTRFCFLKAWTSIARSGNNDESFLANGTRPLYDRIIKYPMLDEAYLKRAKVESFNEDYV-----------TQSLAGPSDKLRATFILTRAVINQLKDRVLAQLPTLEYVSSFTVACAYIWSCIAKSRNDKLQLFGFPIDRRARMKPPIPTAYFGNCVGGCAAIAKTNLLIGKEGFITAAKLIGENLHKTLTDYKDGVLKDNDLVSEGMPTTMTWVSGTPKLRFYDMDFGWGKPKKLETVSID----H-NGA-ISINSCKES-NEDL-EIGVCISATQMEDFVHIFDDGL--------------  -----------------------------FGTYSHTVSSADKCEPVPNNTFNYIDVEITLYPV-ND---T---SC--TR--TT--T--TGLSE----------------------S-I-LT---------------------------------------SELTITMNHTDCNPVFREEYFSVL--NKVATSGFFTGENRYQNISKVCT-LN--FE--IK-CNNK------------------------------------------GSSFKQLTKAKNDDGM----M-S----------------------------------------------------------------------------------------------------------------------------------H-SETV--T-LAG-D---CLSSVDIYILYSNTNAQDYETDTISYRVGNVLDDDSHMPGSCN----------------IHKPITNSKPTRFL | |  | | PVSVDG--ETLTVEAVRRVAEERATVDVPAESIAKAQKSREIFEGIAEQNIPIYGVTTGYGEMIYMQVDKSKEVELQTNLVRSHSAGVGPLFAEDEARAIVAARLNTLAKGHSAVRPIILERLAQYLNEGITPAIPEIGSLGDLAPLSHVASTLIGEGYVLRDGRPVETAQVLAERGIEPLELRFKEGLALINGTSGMTGLGSLVVGRALEQAQQAEIVTALLIEAVRGSTSP----FLAEGHDIARPHEGQIDTAANMRALMRGSGLTVEHADLRRELQKDKEAGKDVQRSEIYLQKAYSLRAIPQVVGAVRDTLYH------------------ARHKLRIELNSANDNPLFFEGKEIFHGANFHGQPIAFAMDFVTIALTQLGVL-----------------------------AERQINRVLNRHLSYGLPEFLVSGDPGLHSGFAGAQYP---ATALVAE--NRTIGPASTQSVPSNGDNQDVVSMG-LIS-ARN-ARRVLSNNNKILAVEY-LAAAQAVDISGRFDGLSPAAKATYEAVRRLVPTLGVDRY-----MADDIELVADALSRGEFLRAIARETDIQLR-  FGT---YSHTVSSADK----------------------------------------------------------------------------------C---EP-------------------------------------------------------------------------------------------VPNN--TF-NY--I--DV-E--IT--LY-PV-N-----DTSCTRT--TT---TG-LS--ES--IL--TS-E--L--------------------------------TI-TM-NH-TDC-NPVFREEYFSVLNKVATSGFFTGENRY--------------------------------QN--ISKVC-TL-NFEIKCNNKGSSFKQLTKAKNDDGMMSHSETVTLA--G------DCLS---S-----------------VDIY------ILY------------------------SN--TNA-QDYE-TDTI--S--YRVGNVL------------------------------------DDDSHMP--GS-C-NI--H-KPI-T-NS--KPTRFL | |  | | QKDLADYPVKLKAYEDEQASIKAALAELEKHKNEDGNLTEPSAQNLVYDLEPNANLSLTTDGKFLKASAVD--DAFSK-STSKAKYVQKILQL------DDLDITNLEQSNDVASSELYGNFGDKAGWSTT---VSNNSQV----------KW-GSVLLERGQSATATYTNLQNSYYNGKKISKIVYKYTVDPKSKFQGQKVWLGIFTDP-----TLGVFASA-YTGQVEKNTSIFIKNEFTFYDEDGKPI-------------------------NFDNALLSVASLNR-----EHNSIEAKDYSGKFVKISGSSIGEKNGIYATDTLNFKQGEGGSRWTYKNSQAGSGWDSSDAPNSWYGAGAIKSGPNNHVTVGATSATNVPVSDPVVPGKDNTDGKKPNIWYSLNGKIRAVNVPKVTKEKPTPPV---------  -----------------------------------------------------------------------FGTYSHTVSSADKC--------EPVPNNTFNY----------------------------IDVE------ITLYPVNDTSC-TRTTTTG--LSESILTS-------------ELTITMNHT--D-C---NPVFREEYFSVLNKVATSGFFTGENRYQ-NISKVCTLNFEIKCNNKGS---SFKQLTKAKNDDGMMSHSETVTLAGDC-LSSVDIYILYSNTNAQDYETDTISYRVGNVLD-----DDS---HMPG---S-------------------CNIHKP---------------------------------------------------I----------------------TNSKPTRFL | |  | |  |  |  | | --- | --- | | (a) | Residue pairs that are at a distance of less than 5 Å in the alignment are highlighted in color. Coloring scheme is based on the property of amino acids, where polar are brightly coloured while non-polar residues are colored in dark shade.(more about the colors used) | | (b) | Ranking of proteins is based on TM-score of the structural alignment between Model1 and the PDB structures in our template library. | | (c) | RMSDa is the RMSD between residues that are structurally aligned by TM-align. | | (d) | IDENa is the percentage sequence identity in the structurally aligned region. | | (e) | Cov. represents the coverage of the alignment by TM-align and is equal to the number of structurally aligned residues divided by length of the model. | |
| |  | | --- | | Function Prediction |   |  | | --- | | Predicted EC Numbers |   | Rank | TM-score | RMSDa | IDENa | Cov. | EC No. | EC-Score | PDB Hit | | --- | --- | --- | --- | --- | --- | --- | --- | | 1 | 0.3926 | 4.74 | 0.13 | 0.61 | 3.1.1.3 | 0.6459 | 1ethC | |  | | | | | | | | | 2 | 0.3935 | 4.67 | 0.13 | 0.61 | 3.1.1.3 | 0.6452 | 1gplA | |  | | | | | | | | | 3 | 0.3890 | 4.64 | 0.12 | 0.59 | 3.1.1.3 | 0.6333 | 1rp1A | |  | | | | | | | | | 4 | 0.4012 | 4.71 | 0.11 | 0.62 | 3.1.1.3 | 0.6318 | 2pplA | |  | | | | | | | | | 5 | 0.3960 | 4.56 | 0.11 | 0.60 | 3.1.1.3 | 0.6275 | 1hplA | |  | | | | | | | |  |  |  | | --- | --- | | (a) | Ranking is based on EC-score. | | (b) | RMSDa is the RMSD between models and the PDB structure in the structurally aligned regions by TM-align. | | (c) | IDENa is percentage sequence identity in the structurally aligned region. | | (d) | Cov. represents the coverage of the alignment and is equal to the number of structurally aligned residues divided by length of model. | | (e) | EC-Score is a confidence score for the Enzyme Classification (EC) Number prediction, which is defined based on the C-score of the structure prediction and the TM-score, IDENa and Cov. of the structural alignment by TM-align between the predicted model and the PDB structures. A prediction with a EC-score >1 signify a prediction with high confidence (upto 3 digit numbers of EC) and vice versa (For detail, see Ambrish, Kucukural and Zhang, Large-scale benchmark of structure-based prediction of protein functions, 2008, in preparation). | |
| |  | | --- | | Predicted GO terms |   | Rank | TMscore | RMSDa | IDENa | Cov. | PDB Hit | Associated GO Terms | | --- | --- | --- | --- | --- | --- | --- | | 1 | 0.4012 | 4.71 | 0.11 | 0.62 | 2pplA | 0004806 0003824 0016042 0005576 0016787 0006629 | | 2 | 0.3960 | 4.56 | 0.11 | 0.60 | 1hplA | 0016042 0016787 0005576 0003824 0006629 0004806 | | 3 | 0.3935 | 5.15 | 0.10 | 0.65 | 1tt9A | 0008152 0016740 0005542 0016829 0030412 0044237 0006547 0005737 0030409 0005794 0003824 | | 4 | 0.3926 | 4.62 | 0.10 | 0.60 | 1bu8A | 0004806 0006629 0016787 0016020 0005576 0003824 0016042 | | 5 | 0.3552 | 4.88 | 0.12 | 0.56 | 1fvgA | 0008113 0055114 0019538 0016491 0016671 | | 6 | 0.3958 | 5.33 | 0.09 | 0.67 | 2gi3A | 0016884 0050567 0006412 0016874 | | 7 | 0.3806 | 5.17 | 0.10 | 0.63 | 1wkyA | 0003824 0005975 0043169 0004553 | | 8 | 0.3660 | 5.42 | 0.10 | 0.63 | 2ddrA | 0005576 0016787 0004767 0019835 0019836 | | 9 | 0.3335 | 5.72 | 0.12 | 0.58 | 1a8dA | 0051609 0033264 0008270 0050827 0016787 0008233 0006508 0046872 0009405 0004222 0008237 0005576 | | 10 | 0.3642 | 5.82 | 0.09 | 0.66 | 2d5lA | 0016020 0008236 0004177 0004274 0006508 | |  | | | | | | Consensus Prediction | | | | |  |  | | Rank | GO-Score | GO Terms |  | | | | 1 | 0.5 | 0005576 |  | | | | 2 | 0.5 | 0016787 |  | | | | 3 | 0.5 | 0003824 |  | | | | 4 | 0.3 | 0004806 |  | | | | 5 | 0.3 | 0016042 |  | | | | 6 | 0.3 | 0006629 |  | | | | 7 | 0.2 | 0016020 |  | | | | 8 | 0.2 | 0006508 |  | | | | 9 | 0.1 | 0055114 |  | | | | 10 | 0.1 | 0004767 |  | | |     |  |  | | --- | --- | | (a) | Ranking in the first table is based on a function prediction score, which is calculated based on the C-score of the structure prediction and the TM-score, IDENa and Cov. of the structural alignment by TM-align between the predicted model and the PDB structures (For detail, see Ambrish, Kucukural and Zhang, Large-scale benchmark of structure-based prediction of protein functions, 2008, in preparation). | | (b) | RMSDa is the RMSD between models and the PDB structure in the structurally aligned regions by TM-align. | | (c) | IDENa is the percentage sequence identity in the structurally aligned region. | | (d) | Cov. represents the coverage of the alignment and is equal to the number of structurally aligned residues divided by length of model. | | (e) | GO-Score in the second table is defined as a relative frequency of the GO terms appearing in the top 10 functional homologs. A prediction with a GO-score >0.6 signify a prediction with high confidence and vice versa. | |
| |  | | --- | | Predicted Binding Site |   | |  | | **Predicted Binding Site Residues:**   |  |  |  |  |  |  |  |  |  |  |  |  |  |  |  | | --- | --- | --- | --- | --- | --- | --- | --- | --- | --- | --- | --- | --- | --- | --- | | HIS | :6( 0.14) |  | VAL | :8( 0.14) |  | THR | :30( 1.28) |  | THR | :43( 0.10) |  | THR | :45( 0.10) |  | | GLY | :46( 0.10) |  | PHE | :74( 1.28) |  | LYS | :110( 1.40) |  | SER | :112( 1.40) |  | SER | :113( 1.40) |  | | PHE | :114( 1.40) |  | LEU | :135( 0.10) |  | | **Identified functional homologs:**  | Rank | PDB Hit | TM-score | RMSDa | IDENa | Cov. | BSscore | | --- | --- | --- | --- | --- | --- | --- | | 1 | 1ethA | 0.3926 | 4.74 | 0.13 | 0.59 | 3.06 | | 2 | 1gpl\_ | 0.3935 | 4.67 | 0.13 | 0.59 | 3.05 | | 3 | 1rp1\_ | 0.3890 | 4.64 | 0.12 | 0.58 | 2.85 | | 4 | 2pplA | 0.4012 | 4.71 | 0.11 | 0.60 | 2.84 | | 5 | 2pc8A | 0.3859 | 4.98 | 0.12 | 0.59 | 2.82 | | 6 | 1hplA | 0.3960 | 4.56 | 0.11 | 0.58 | 2.76 | | 7 | 1lpaB | 0.3934 | 4.61 | 0.11 | 0.58 | 2.74 | | 8 | 1lpbB | 0.3870 | 4.96 | 0.11 | 0.60 | 2.59 | | 9 | 1eqcA | 0.3860 | 5.08 | 0.11 | 0.59 | 2.58 | | 10 | 1wkyA | 0.3806 | 5.17 | 0.10 | 0.61 | 2.39 |  |  |  | | --- | --- | | (a) | Binding site residues reported above are derived from a consensus prediction of the top functional homologs (shown in the table). Values shown inside the parenthesis beside each residue reflect the relative frequency with which the predicted residue in the model structurally aligns with known binding site residue in the functional homolog(s). A relative frequency >1 signify a binding site residue prediction with high confidence and vice-versa. | | (b) | Ranking of the identified functional homologs in the table is based on BSscore, which is calculated based on the C-score of the structure prediction and the TM-score, IDENand Cov. a of the structural alignment by TM-align between the predicted model and the PDB structures (For detail, see Ambrish, Kucukural and Zhang, Large-scale benchmark of structure-based prediction of protein functions, 2008, in preparation). | | (c) | RMSDa is the RMSD between residues that are structurally aligned by TM-align. | | (d) | IDENa is the percentage sequence identity in the structurally aligned region. | | (e) | Cov. represents the coverage of the alignment by TM-align and is equal to the number of structurally aligned residues divided by length of the model. | | (f) | Predicted binding site residues are shown in green sphere while N & C terminus in the model are marked by blue and red sphere respectively. | | |

  
  

---

  

|  |  |
| --- | --- |
|  | Please cite following articles when you use the I-TASSER server: |
|
| 1. | Yang Zhang. I-TASSER server for protein 3D structure prediction. BMC Bioinformatics, 9:40 (2008). |
| 2. | Yang Zhang. Template-based modeling and free modeling by I-TASSER in CASP7. Proteins, 8: 108-117 (2007). |
| 3. | Sitao Wu, Jeffrey Skolnick, Yang Zhang. Ab initio modeling of small proteins by iterative TASSER simulations. BMC Biology, 5:17 (2007). |
