# Supplementary material for: SECRET domain of variola virus CrmB protein can be a member of poxviral type II chemokine-binding proteins family
Source: BMC Res Notes. 2010 Oct 27;3:271. doi: 10.1186/1756-0500-3-271 (PMC2987869; doi:10.1186/1756-0500-3-271)
Supplement: Additional file 1 — Output from I-TASSER web-server. This file contains the output from I-TASSER web-server including all generated models and alignments. [file 1756-0500-3-271-S1.ZIP › I-TASSER results_files/color.html]

Colored Alignment
[Home]
[Server]
[Queue]
[About]
[Remove]
[Potential]
[Decoys]

## Coloring scheme used in alignment

  
  

| Amino Acid Name | Single Letter Code | Color |
| --- | --- | --- |
| Glycine | G |  |
| Histidine | H |  |
| Proline | P |  |
| Alanine | A |  |
| Tryptophan | W |  |
| Serine | S |  |
| Threonine | T |  |
| Valine | V |  |
| Isoleucine | I |  |
| Leucine | L |  |
| Phenylalanine | F |  |
| Tyrosine | Y |  |
| Cysteine | C |  |
| Methionine | M |  |
| Aspartate | D |  |
| Glutamate | E |  |
| Asparagine | N |  |
| Glutamine | Q |  |
| Lysine | N |  |
| Arginine | Q |  |
