# Supplementary material for: SECRET domain of variola virus CrmB protein can be a member of poxviral type II chemokine-binding proteins family
Source: BMC Res Notes. 2010 Oct 27;3:271. doi: 10.1186/1756-0500-3-271 (PMC2987869; doi:10.1186/1756-0500-3-271)
Supplement: Additional file 1 — Output from I-TASSER web-server. This file contains the output from I-TASSER web-server including all generated models and alignments. [file 1756-0500-3-271-S1.ZIP › index.html]

The fact that SECRET
proteins lack substantial amino acid sequence homology to other known proteins
was repeatedly highlighted in numerous papers in the field. However we also did
BLAST, PSI-BLAST searches with the sequence of VARV-CrmB SECRET domain and were
unable to find proteins with significant level of homology (excepting sequences
of orthologous proteins of other poxviruses). The sequence of the SECRET domain
was classified as DUF1406
(PF07190) with NCBI Conservative Domains Database and this domain had no known
function, no known structures and no other proteins than poxviral.

After our modeling task
was submitted to the I-TASSER, the server generated several models and 4 of top
5 models shared similar structure with minor distinctions, and we chose the
best model on the basis of its I-TASSER C-score. The I-TASSER output could be still
found at http://zhang.bioinformatics.ku.edu/I-TASSER/output/S28327/
and is included in the supplement with all
files (including the generated models).

We
also modeled SECRET structure with Modeller
(9v2). Alignment of the SECRET and vCCI was redacted according to their
secondary structures (either extracted from 1CQ3 for vCCI or predicted with
PSIPRED for the SECRET), and used as input for Modeller. The model
was similar to that produced by I-TASSER. RMSD between 176 aligned Ca atoms of the models
was about 3.4 angstroms and RMSD between the Ca atoms of the best
fitted 125 residues was about 1.5 angstroms.
