# Supplementary figures and images for: SECRET domain of variola virus CrmB protein can be a member of poxviral type II chemokine-binding proteins family
Source: BMC Res Notes. 2010 Oct 27;3:271. doi: 10.1186/1756-0500-3-271 (PMC2987869; doi:10.1186/1756-0500-3-271)

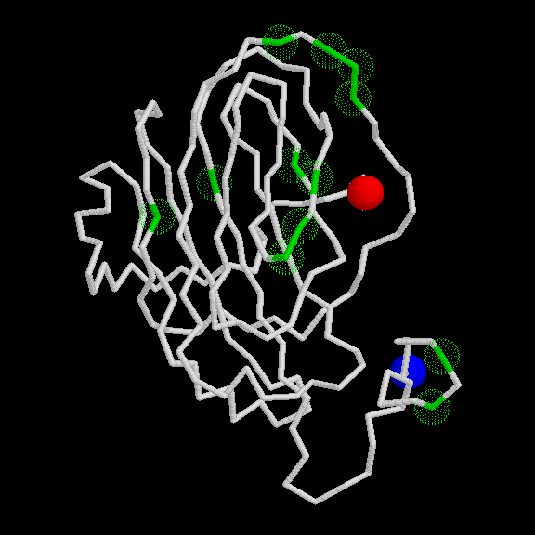

Supplement: Additional file 1 — Output from I-TASSER web-server. This file contains the output from I-TASSER web-server including all generated models and alignments. [file 1756-0500-3-271-S1.ZIP › I-TASSER results_files/bsite.gif]

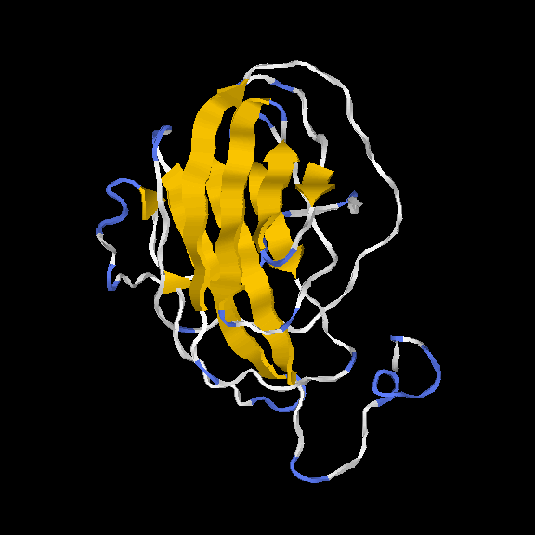

Supplement: Additional file 1 — Output from I-TASSER web-server. This file contains the output from I-TASSER web-server including all generated models and alignments. [file 1756-0500-3-271-S1.ZIP › I-TASSER results_files/model1.gif]

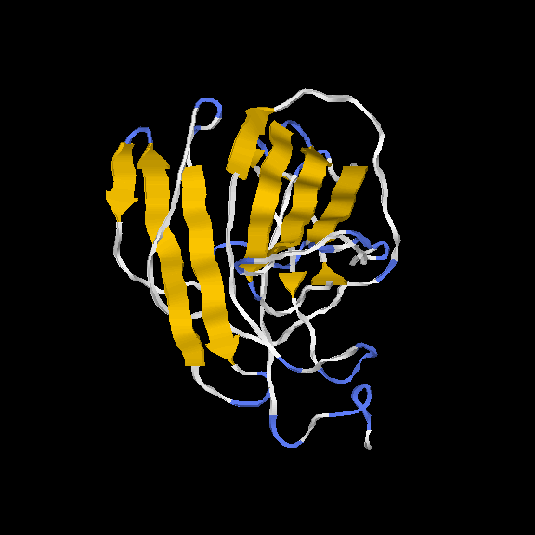

Supplement: Additional file 1 — Output from I-TASSER web-server. This file contains the output from I-TASSER web-server including all generated models and alignments. [file 1756-0500-3-271-S1.ZIP › I-TASSER results_files/model2.gif]

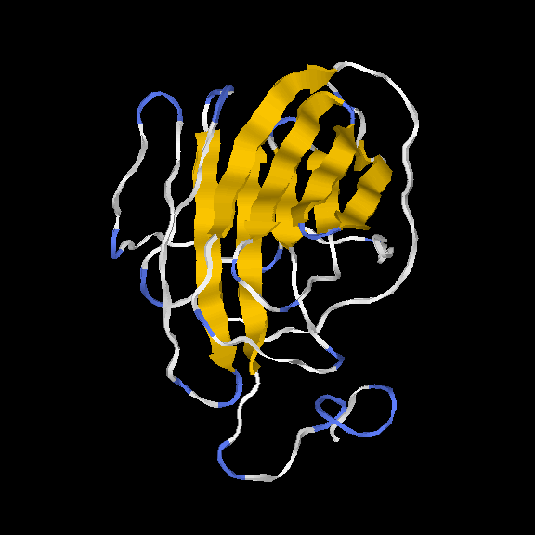

Supplement: Additional file 1 — Output from I-TASSER web-server. This file contains the output from I-TASSER web-server including all generated models and alignments. [file 1756-0500-3-271-S1.ZIP › I-TASSER results_files/model3.gif]

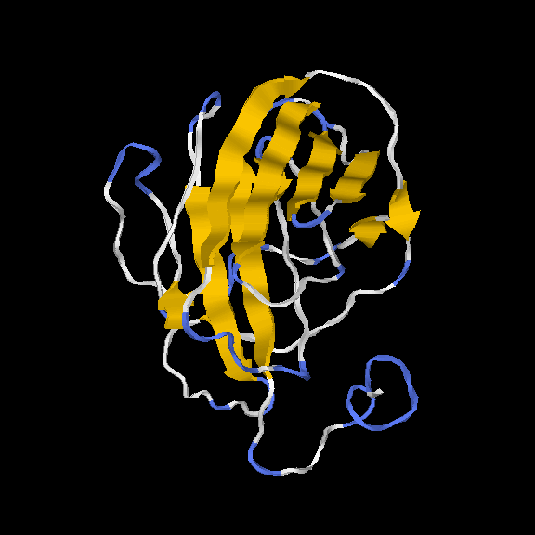

Supplement: Additional file 1 — Output from I-TASSER web-server. This file contains the output from I-TASSER web-server including all generated models and alignments. [file 1756-0500-3-271-S1.ZIP › I-TASSER results_files/model4.gif]

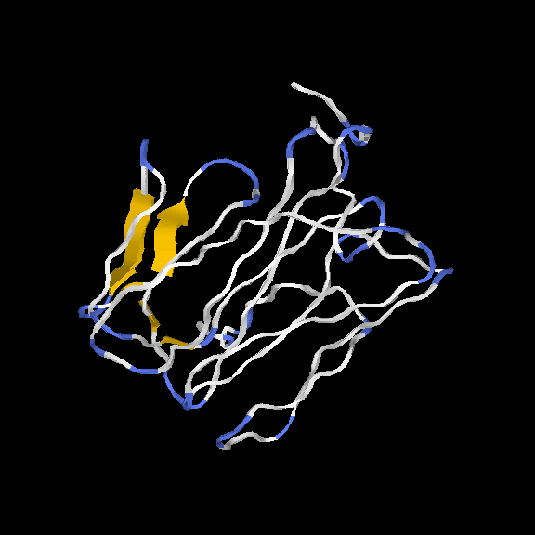

Supplement: Additional file 1 — Output from I-TASSER web-server. This file contains the output from I-TASSER web-server including all generated models and alignments. [file 1756-0500-3-271-S1.ZIP › I-TASSER results_files/model5.gif]
